# Municipal sewage sludge compost promotes *Mangifera persiciforma* tree growth with no risk of heavy metal contamination of soil

**DOI:** 10.1038/s41598-017-13895-y

**Published:** 2017-10-17

**Authors:** Shuangshuang Chu, Daoming Wu, Liyin L. Liang, Fengdi Zhong, Yaping Hu, Xinsheng Hu, Can Lai, Shucai Zeng

**Affiliations:** 1 College of Forestry and Landscape Architecture, South China Agricultural University, Guangzhou, 510642 China; 2 School of Science and Environmental Research Institute, University of Waikato, Private Bag 3105, Hamilton, 3240 New Zealand

## Abstract

Application of sewage sludge compost (SSC) as a fertilizer on landscaping provides a potential way for the effective disposal of sludge. However, the response of landscape trees to SSC application and the impacts of heavy metals from SSC on soil are poorly understood. We conducted a pot experiment to investigate the effects of SSC addition on *Mangifera persiciforma* growth and quantified its uptake of heavy metals from SSC by setting five treatments with mass ratios of SSC to lateritic soil as 0%:100% (CK), 15%:85% (S15), 30%:70% (S30), 60%:40% (S60), and 100%:0% (S100). As expected, the fertility and heavy metal concentrations (Cu, Zn, Pb and Cd) in substrate significantly increased with SSC addition. The best performance in terms of plant height, ground diameter, biomass and N, P, K uptake were found in S30, implying a reasonable amount of SSC could benefit the growth of *M. persiciforma*. The concentrations of Cu, Pb and Cd in S30 were insignificantly different from CK after harvest, indicating that *M. persiciforma* reduced the risk of heavy metal contamination of soil arising from SSC application. This study suggests that a reasonable rate of SSC addition can enhance *M. persiciforma* growth without causing the contamination of landscaping soil by heavy metals.

## Introduction

Municipal sewage treatment projects often produce a considerable amount of sludge each year across the world^1^, especially in developing countries^2^. China produces more than 30 million tons of municipal sewage sludge annually, with a yearly increase in excess of 10% in recent decades^3^. Sewage sludge disposal has become a significant challenge in environmental management. Sewage sludge could be used as an organic fertilizer due to its richness in nutrients and organic matter^4,5^. However, the potential transmission of heavy metals in sewage sludge through the food chain limits its application to farming systems^6–8^. As an alternative, sewage sludge application as a fertilizer on landscaping could have obvious advantages, including a larger area of applicability and potentially higher application rates^9–11^. Moreover, sewage sludge application exhibits better effects on growth for several landscape plants^12^ than do traditional organic fertilizers. This application could provide an effective means of municipal sewage sludge disposal.

Instead of applying the smelly fresh sewage sludge with larger amounts of heavy metals as landscaping fertilizers, using sewage sludge compost (SSC) could be more advantageous since SSC has less odorous emission and likelihood of the bioleaching of heavy metals^13^. However, an overdose of SSC will increase the heavy metal contents in plants and further inhibit plant growth^14^. Previous studies found that SSC application increased the accumulation of Cd and Pb in *Swietenia mahagoni* ^15^ and increased the concentration of Cd, Cr and Pb in *Morus alba* ^14^. Moreover, heavy metals from SSC may remain in the soil and cause soil pollution by too heavy application^16^. For example, Yeganeh *et al*.^17^ noted that compared to the control, the Zn, Cu, and Pb concentrations in soils continually amended with sludge for four years are 1600, 7 and 4.5 times higher, respectively. The heavy metals accumulation could leach from soil to groundwater under reducing conditions^18^. Therefore, effective measures should be introduced to reduce the phytotoxicity and ecological risk of heavy metals caused by the recycling of SSC to landscape soil.

As reviewed by Liu^19^, toxic effects were often caused by high concentrations of heavy metals in SSC and unrealistically high amounts of SSC amendments. This suggested that controlling the amount of sludge application may be an effective way to reduce the phytotoxicity of the heavy metals. However, the application dosage of SSC varies across plant species since their tolerances to heavy metals are different. Kumar and Chopra^20^ suggested that the application amount of SSC to *Phaseolus vulgaris* as a fertilizer needed to be controlled below 40%. De Lucia *et al*.^21^ suggested that a 30% compost level of SSC guaranteed the best performance for *Rhamnus* and *Myrthus* plants, and a 45% compost level for *Phillyrea* plants.

That being said, the planting of suitable species is an effective way to reduce heavy metals for SSC utilization in landscaping. Belhaj *et al*.^22^ noted that *Helianthus annuus* planted on soils amended with SSC had improved growth and helped to remove the heavy metals from the soil. Majid *et al*.^23,24^ found that Cu, Zn and Pb in a sawdust sludge-contaminated soil were significantly reduced by *Fagopyrum dibotrys* and *Pluchea indica*. Some wetland plants, such as *Cyperus alternifolius*, *Colocasia gigantea*, and *Iris pseudacorus*, can grow in sludge and accumulate heavy metals in their tissues^25^. To date, the most reported heavy-metal hyperaccumulators are herbaceous plants with a limited biomass. Their low biomass limits the amount of uptake of heavy metals from soil although the above-ground parts of herbaceous plants can accumulate heavy metals at a high level^26^. Woody plants could be better candidates for removing heavy metals in sludge compost due to their apparent advantages, including hyperaccumulation, rapid growth, large biomass, well-developed root systems, and long lives^27,28^. Previous studies indicated that woody plants, such as *Salix* spp.^29,30^ and *Populus* spp.^31^, were well adapted to SSC and were effective in taking up the heavy metals in sludge. These results suggested that selecting suitable woody plants that have high biomass and heavy metals tolerance can be an effective way to ensure the ecological safety of SSC utilization as soil amendments in landscaping.

*Mangifera persiciforma* C.Y. Wu et T.L. Ming is a common landscape tree in the tropics and subtropics. It grows rapidly and is adaptable to various soil conditions. The species is now a very popular landscape tree and is widely planted in urban areas in South China^32^. If *M. persiciforma* grows well in SSC-amended soil and is able to take up a desirable amount of heavy metals, landscaping with the application of SSC would become practical in the tropics and subtropics. However, the growth response of *M. persiciforma* to SSC and the fate of heavy metals in the SSC are still poorly understood.

Here, we conducted a glasshouse experiment using mixed SSC and urban soil at different mass ratios and observing the growth response of *M. persiciforma*. First, we analysed the changes in the physicochemical properties of the soil under different application amounts of SSC. The effects of SSC application on *M. persiciforma* growth were then observed and the relevant mechanisms were further studied by measuring the concentrations of nutrients and heavy metals in *M. persiciforma*. Last, the potential environmental risk of heavy metals in SSC-amended soils after planting *M. persiciforma* were assessed.

## Results

### Effect of SSC Application Rates on the Physicochemical Properties of the Substrate

As expected, the control (CK) had the lowest value of porosity, pH, nutrient contents and heavy metal concentrations. The SSC (S100) had the highest values (Table 1). The application of SSC changed the physicochemical properties of the substrates (Table 1). The bulk density decreased with increasing amounts of SSC, while pH increased 0.51–1.64 unit after SSC application. The application of SSC obviously triggered the moisture retention capacity of lateritic soil by increasing capillary capacity and aeration porosity. The concentrations of organic matter and nutrients as well as heavy metals increased with an increase in the SSC amendment ratios. However, when the amount of applied SSC was less than 60%, the heavy metal concentrations in the substrates are lower than the tier II level (Cu < 50 mg·kg^−1^, Zn < 200 mg·kg^−1^, Pb < 250 mg·kg^−1^, and Cd < 0.3 mg·kg^−1^) of the “Environmental Quality Standards for Soil of China” (GB 15618–1995)^33^.

**Table 1:** Basic physiochemical properties of the substrate in different treatments (mean ± SE, n = 5).

Physicochemical properties	Treatments				
	CK	S15	S30	S60	S100
Bulk density (g·cm^−3^)	1.41 ± 0.01a	1.39 ± 0.11a	1.23 ± 0.32a	1.17 ± 0.35b	0.87 ± 0.01c
Aeration porosity (%)	46.62 ± 1.45c	47.57 ± 1.42c	48.81 ± 0.50b	56.10 ± 0.42b	67.33 ± 1.38a
Capillary capacity(g·kg^−1^)	215.43 ± 4.55d	236.57 ± 9.02c	249.59 ± 4.99bc	264.46 ± 0.80b	283.57 ± 6.24a
pH	5.01 ± 0.01e	5.52 ± 0.02d	5.90 ± 0.03c	6.48 ± 0.02b	6.65 ± 0.01a
Organic matter (g·kg^−1^)	6.47 ± 0.78d	14.71 ± 0.28dc	26.69 ± 0.45c	47.50 ± 2.99b	172.87 ± 9.97a
Total N (g·kg^−1^)	0.13 ± 0.01e	0.55 ± 0.01d	1.28 ± 0.03c	3.96 ± 0.02b	12.13 ± 0.28a
Available N (mg·kg^−1^)	44.62 ± 0.45d	63.52 ± 2.50d	173.24 ± 4.17c	571.48 ± 6.67b	1663.51 ± 27.69a
Total P (g·kg^−1^)	1.45 ± 0.04e	7.64 ± 0.44d	13.69 ± 0.61c	25.52 ± 0.92b	54.73 ± 1.22a
Available P (mg·kg^−1^)	0.57 ± 0.02e	15.88 ± 0.72d	20.25 ± 0.5c	37.58 ± 0.97b	120.09 ± 3.38a
Total K (g·kg^−1^)	4.51 ± 0.20e	8.93 ± 0.93d	16.28 ± 0.34c	18.61 ± 0.32b	22.30 ± 0.22a
Available K (mg·kg^−1^)	49.71 ± 1.47e	81.01 ± 3.18d	182.06 ± 4.59c	352.97 ± 6.71b	374.15 ± 8.61a
Cu (mg·kg^−1^)	9.38 ± 0.06d	13.55 ± 0.52cd	17.84 ± 1.00c	50.92 ± 2.96b	162.96 ± 4.76a
Zn (mg·kg^−1^)	31.37 ± 1.39e	93.50 ± 7.12d	163.88 ± 6.57c	318.32 ± 20.83b	742.77 ± 38.31a
Pb (mg·kg^−1^)	33.27 ± 2.34d	38.83 ± 2.00d	49.58 ± 2.76c	59.91 ± 1.48b	89.49 ± 2.95a
Cd (mg·kg^−1^)	0.14 ± 0.00d	0.17 ± 0.00d	0.24 ± 0.00c	0.85 ± 0.02b	2.59 ± 0.04a

Note Data with unshared letters in the same row are significantly different (Duncan’s test, α = 0.05). Treatments include: CK, 100% lateritic soil; S15, 15% SSC and 85% lateritic soil; S30, 30% SSC and 70% lateritic soil; S60, 60% SSC and 40% lateritic soil; S100, 100% SSC.

### Effects on Plant Height, Ground Diameter and Biomass

#### Plant Height and Ground Diameter

**Figure 1:**
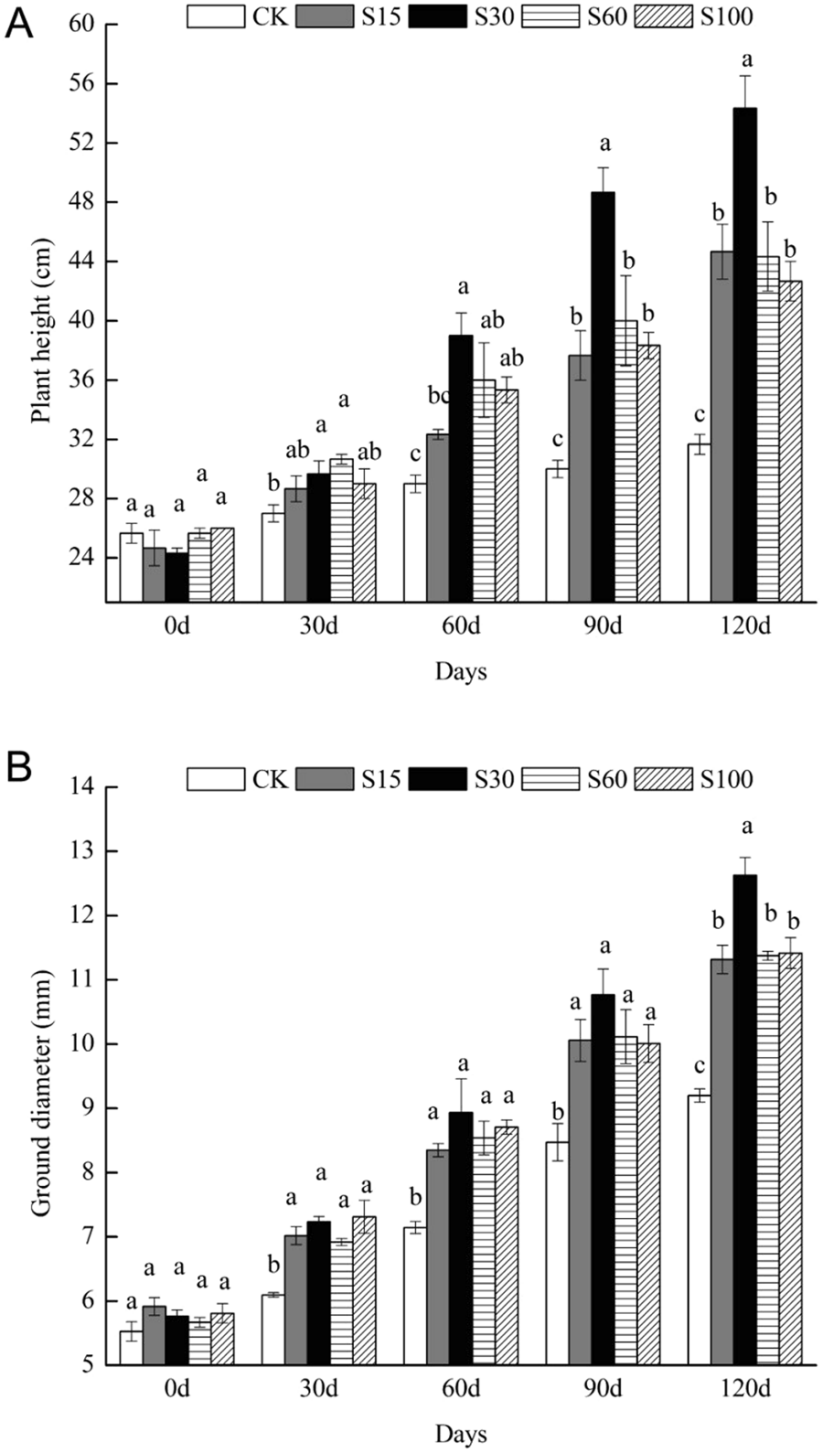
Height (**A**) and ground diameter (**B**) of *M. persiciforma* under different treatments. Data are shown as the mean ± SE from the five replicates of each treatment. For each group, columns with the same letters are not significantly different (α = 0.05 by Duncan’s test).

Compared to CK, the treatments with SSC application had different promoting effects on plant height and ground diameter (Fig. 1A and B). After 30 d of growth, the plant height under S30 and S60 treatments significantly increased compared to CK (*P* < *0.05*), but no significant differences were found with S15 and S100 (*P* > *0.05*). After 60 d, the height of *M. persiciforma* in S30, S60 and S100 was significantly higher than CK (*P* < *0.05*), but S15 was still not significantly different from CK (*P* > *0.05*). After 90 d, the seedling heights of the treatments with SSC application were significantly greater than CK (*P* < *0.05*) and the best effect was observed in S30, which had a significantly higher plant height than the other treatments (*P* < *0.05*) (Fig. 1A). This trend did not change at the end of the pot experiment.

SSC significantly increased the ground diameters relative to CK after 30 d of growth (*P* < *0.05*), but a significant difference was observed among the treatments with SSC application only at the end of the experiment (*P* < *0.05*), and the highest ground diameter also appeared in S30 (Fig. 1B).

#### Biomass

S15 and S30 significantly increased the biomass of roots, shoots, leaves and total plant of *M. persiciforma* compared to CK (*P* < *0.05*) (Table 2). S30 was superior to S15 for *M. persiciforma* leaves and total plant biomass. The highest values of the dry weight of total plant biomass was observed in S30 (52.40 g·plant^−1^), being significantly different from the other treatments (*P* < *0.05*). The biomass of stem, leaf, and total plant of S60 and S100 were not significantly different from those of the CK, and the root biomass in S100 was significantly less than that of CK (*P* < *0.05*).

**Table 2:** Biomass of *M. persiciforma* in different treatments (g·plant^−1^, dry weight) (mean ± SE, n = 5).

Treatments	Tissues			Total plant
	Root	Stem	Leaf	
CK	11.63 ± 0.21b	12.58 ± 0.47c	12.12 ± 0.32 cd	36.33 ± 0.55c
S15	12.91 ± 0.17a	15.53 ± 0.37ab	17.36 ± 0.40b	45.79 ± 0.63b
S30	13.52 ± 0.58a	17.87 ± 1.53a	21.01 ± 1.44a	52.40 ± 3.29a
S60	10.99 ± 0.23bc	13.79 ± 0.53bc	14.19 ± 0.24c	38.97 ± 0.74c
S100	10.31 ± 0.21c	12.70 ± 0.45c	11.20 ± 0.92d	34.20 ± 1.53c

Note Data followed by different letters in the same column are significantly different (Duncan’s test, α = 0.05). Treatments include: CK, 100% lateritic soil; S15, 15% SSC and 85% lateritic soil; S30, 30% SSC and 70% lateritic soil; S60, 60% SSC and 40% lateritic soil; S100, 100% SSC.

### N, P, and K Concentrations and Storage in *M. persiciforma*

#### N, P, and K Concentrations

The N, P, and K concentrations were significantly higher in the roots, shoots, leaves of *M. persiciforma* growing in the treatments with SSC application compared with the control (*P* < *0.05*) (Table 3). The highest values for total plant N and P concentrations (19.20 and 2.48 g·kg^−1^, respectively) were observed in the S30 treatment, whereas the lowest values were observed in CK (11.98 and 0.89 g·kg^−1^, respectively). The K concentration in the total plant increased with the SSC application rate, and the highest value was observed in S100 (16.56 g·kg^−1^), significantly higher than those of S15 and S30, but not significantly different from S60 (*P* > *0.05*).

**Table 3:** Nutrient concentrations (g·kg^−1^ dry matter) in different tissues of *M. persiciforma* (mean ± SE, n = 5).

Elements	Tissues	Treatments				
		CK	S15	S30	S60	S100
N	Root	10.71 ± 0.45c	17.52 ± 0.74a	17.83 ± 0.55a	15.76 ± 0.50b	15.60 ± 0.36b
	Stem	10.30 ± 0.20c	13.68 ± 0.13b	16.67 ± 0.47a	15.96 ± 0.43a	13.86 ± 0.45b
	Leaf	14.97 ± 0.25d	19.46 ± 0.54b	22.27 ± 0.32a	19.26 ± 0.28b	17.19 ± 0.08c
	Total plant	11.98 ± 0.19d	16.96 ± 0.16b	19.20 ± 0.14a	17.12 ± 0.08b	15.47 ± 0.27c
P	Root	0.88 ± 0.05b	1.34 ± 0.16a	1.59 ± 0.05a	1.46 ± 0.07a	1.35 ± 0.20a
	Stem	0.57 ± 0.03c	1.31 ± 0.07b	2.46 ± 0.22a	1.74 ± 0.19b	1.47 ± 0.12b
	Leaf	1.23 ± 0.04c	1.87 ± 0.04b	3.06 ± 0.26a	2.70 ± 0.29b	2.11 ± 0.09b
	Total plant	0.89 ± 0.03d	1.53 ± 0.05c	2.48 ± 0.03a	2.01 ± 0.14b	1.64 ± 0.10c
K	Root	12.83 ± 0.68b	19.32 ± 2.15a	18.62 ± 0.40a	18.64 ± 0.92a	17.92 ± 0.42a
	Stem	9.26 ± 0.15d	11.43 ± 0.55c	13.88 ± 0.60b	15.90 ± 0.23a	16.58 ± 0.81a
	Leaf	8.54 ± 0.33c	10.51 ± 0.21b	13.53 ± 0.80a	14.66 ± 0.81a	15.27 ± 0.55a
	Total plant	10.16 ± 0.34d	13.40 ± 0.47c	14.93 ± 0.38b	16.22 ± 0.55ab	16.56 ± 0.58a

Note: Data followed by different letters in the same row are significantly different (Duncan’s test, α = 0.05). Treatments include: CK, 100% lateritic soil; S15, 15% SSC and 85% lateritic soil; S30, 30% SSC and 70% lateritic soil; S60, 60% SSC and 40% lateritic soil; S100, 100% SSC.

#### N, P, and K Storages

**Figure 2:**
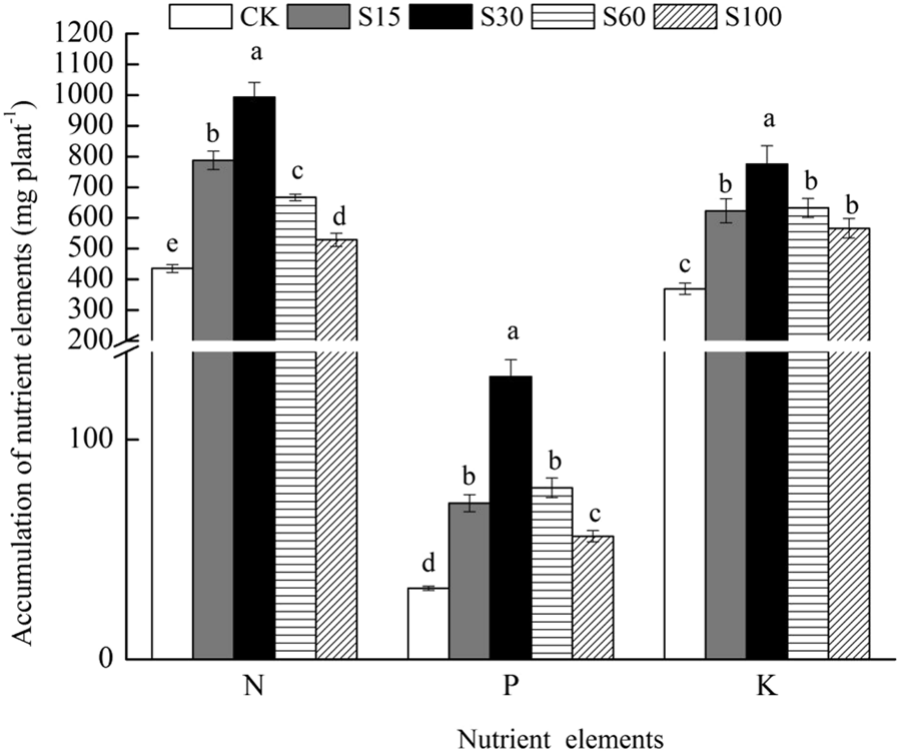
Accumulation of nutrients by *M. persiciforma* under different treatments. Data are shown as the mean ± SE from the five replicates of each treatment. For each group, columns with the same letters are not significantly different (α = 0.05 by Duncan’s test).

N, P and K storages in *M. persiciforma* in treatments with SSC application were significantly higher than those in CK (*P* < *0.05*) (Fig. 2). Significant differences in N storage were observed across treatments, and the storage order was S30 > S15 > S60 > S100. For P storage, the maximum values occurred in S30 and were significantly different from S15, S60 and S100, while no significant differences existed between S15 and S60 (*P* > *0.05*), but the two treatments were significantly higher than S100 (*P* < *0.05*). No significant differences were found for K storage in S15, S60 and S100 (*P* > *0.05*), but they were significantly lower than S30.

Therefore, S30 showed the best performance in terms of the storage of N, P and K in *M. persiciforma*, which were significantly higher than in other treatments and were 2.28, 3.97 and 2.09 times higher than that in CK, respectively.

### Heavy Metals Accumulation in *M. persiciforma*

#### Heavy Metal Concentrations in Different Plant Parts

The heavy metal concentrations in *M. persiciforma* plants are shown in Table 4. Cu concentrations in the roots and total plants for all treatments were significantly higher than the control, but S30, S60, and S100 had insignificant effects on Cu concentrations in stems and leaves compared to CK (*P* > *0.05*). S100 had significantly higher Cu concentrations in the stem, leaves and total plant than other treatments (*P* < *0.05*). The Zn concentrations in roots, stems, leaves and total plant increased with the SSC rate, and S100 had significantly higher Zn concentration than all other treatments (*P* < *0.05*). Interestingly, no significant differences were found in the Pb concentration in either roots, stems, leaves or total plant of *M. persiciforma* among the treatments (*P* > *0.05*). The addition of SSC had no significant effect on the Cd concentrations in leaves with comparison to CK (*P* > *0.05*), while S30, S60, and S100 significantly increased Cd concentrations in the roots, and the rate of 100% had significantly higher Cd concentrations in the stems and total plant compared with other treatments (*P* < *0.05*).

**Table 4:** Heavy metal concentrations (mg·kg^−1^ dry matter) in roots, stems, leaves and total plant of *M. persiciforma* (mean ± SE, n = 5).

Heavy metals	Tissue	Treatments				
		CK	S15	S30	S60	S100
Cu	Root	10.60 ± 0.74Ac	13.84 ± 1.00Ab	15.39 ± 0.10Ab	18.74 ± 0.34Aa	20.39 ± 1.22Aa
	Stem	3.91 ± 0.49Bb	4.09 ± 0.04Bb	4.32 ± 0.23Cb	4.54 ± 0.08Cb	5.83 ± 0.95Ba
	Leaf	4.80 ± 0.47Bb	4.91 ± 0.11Bb	5.17 ± 0.34Bb	5.35 ± 0.19Bb	6.91 ± 0.75Ba
	Total plant	6.33 ± 0.26d	7.24 ± 0.31c	7.45 ± 0.32c	8.84 ± 0.10b	10.55 ± 0.36a
Zn	Root	20.24 ± 0.91Ad	62.09 ± 2.47Ac	77.17 ± 1.72Ac	128.39 ± 10.35Ab	186.72 ± 5.02Aa
	Stem	11.15 ± 0.70Cd	13.58 ± 0.56Bc	23.18 ± 2.07Bc	49.40 ± 1.34Bb	77.71 ± 3.86Ba
	Leaf	14.30 ± 1.11Bc	15.06 ± 2.99Bc	18.06 ± 1.77Bc	27.16 ± 3.61Cb	36.02 ± 3.82Ca
	Total plant	15.12 ± 0.46e	28.26 ± 0.50d	34.70 ± 1.71c	63.60 ± 3.58b	96.96 ± 1.70a
Pb	Root	11.14 ± 2.52Aa	11.85 ± 0.98Aa	11.93 ± 1.16Aa	10.95 ± 3.29Aa	10.63 ± 2.61Aa
	Stem	1.31 ± 0.20Ba	1.73 ± 0.08Ba	2.39 ± 0.50Ba	1.83 ± 0.16Ba	1.78 ± 0.55Ba
	Leaf	1.16 ± 0.08Ba	1.75 ± 0.80Ba	1.86 ± 0.33Ba	1.14 ± 0.13Ba	1.09 ± 0.20Ba
	Total plant	4.37 ± 0.73a	4.69 ± 0.14a	4.54 ± 0.14a	4.15 ± 1.00a	4.24 ± 0.92a
Cd	Root	0.22 ± 0.03Ad	0.35 ± 0.03Acd	0.43 ± 0.06Abc	0.53 ± 0.06Aab	0.58 ± 0.05Aa
	Stem	0.05 ± 0.01Bb	0.08 ± 0.01Bb	0.08 ± 0.03Bb	0.09 ± 0.03Bb	0.22 ± 0.03Ba
	Leaf	0.04 ± 0.01Ba	0.05 ± 0.01Ba	0.06 ± 0.04Ba	0.06 ± 0.04Ba	0.09 ± 0.00Ca
	Total plant	0.10 ± 0.01c	0.15 ± 0.01bc	0.16 ± 0.03bc	0.21 ± 0.02b	0.29 ± 0.02a

Note: Means with different upper-case letters are significantly different among tissues for the same treatment and same heavy metal (α = 0.05 by Duncan test). Means in a row with different lower-case letters are significantly different among treatments (α = 0.05 by Duncan test). Treatments include: CK, 100% lateritic soil; S15, 15% SSC and 85% lateritic soil; S30, 30% SSC and 70% lateritic soil; S60, 60% SSC and 40% lateritic soil; S100, 100% SSC.

In all the treatments, the roots showed significantly higher heavy metal concentrations than the stems and leaves, and the highest concentration of heavy metals in the roots of *M. persiciforma* reached 20.39 mg·kg^−1^ (Cu), 186.72 mg·kg^−1^ (Zn), 11.93 mg·kg^−1^ (Pb) and 0.58 mg·kg^−1^ (Cd), respectively.

#### Heavy Metals Accumulation

**Figure 3:**
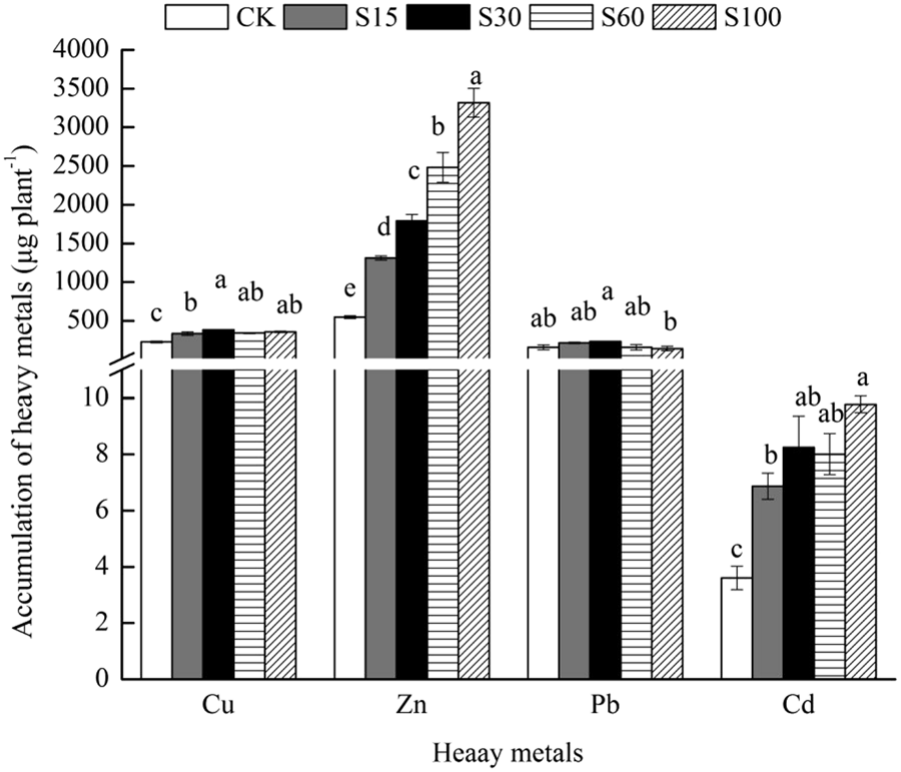
Accumulation of heavy metals by *M. persiciforma* under different treatments. Data are shown as the mean ± SE from the five replicates of each treatment. For each group, columns with the same letters are not significantly different (α = 0.05 by Duncan’s test).

SSC addition significantly increased the accumulation of Cu, Zn and Cd in *M. persiciforma* compared to CK (*P* < *0.05*) (Fig. 3). The highest accumulation of Cu was found in S30. The accumulation of Zn in *M. persiciforma* increased significantly with SSC amount (*P* < *0.05*). The accumulation of Pb in treatments with SSC application did not differ from that of CK, but S30 had significantly higher Pb accumulation than S100. The accumulation of Cd generally increased with the SSC application rate, with that of S100 being significantly higher than CK and S15 (*P* < *0.05*). The accumulation of metals was in the order of Zn > Cu > Pb > Cd in each treatment.

#### Changes of Heavy Metals in Substrates after Seedling Harvest

The concentrations of Cd, Pb, Cu and Zn in the substrates after *M. persiciforma* harvest decreased by a different extent (i.e., 12.7–33.4% for Cu, 11.8–38.7% for Zn, 13.2–22.4% for Pb, and 11.6–22.9% for Cd). The greatest rates of decline in Cu, Pb and Cd concentrations in the substrate occurred in S30, and that of Zn occurred in S15.

After seedling harvest, S15 had no significant Cu, Zn, Pb and Cd residues compared to CK. The concentrations of Cu, Pb and Cd in the substrate of S30 were not significantly different from CK after harvest, while they were significantly higher than CK before planting (*P* < *0.05*). The residual heavy metal concentrations in S60 were 40.02 (Cu), 239.09 (Zn), 48.58 (Pb) and 0.71 mg·kg^−1^ (Cd), still significantly higher than CK (*P* < *0.05*), but Cu, Zn and Pb did not exceed the tier II level (Cu < 50 mg·kg^−1^, Zn < 200 mg·kg^−1^ and Pb < 250 mg·kg^−1^) of the “Environmental Quality Standards for Soil of China” (GB 15618–1995)^33^. However, the heavy metals concentrations in S100 were still higher than the GB standard.

## Discussion

The benefits of using SSC as a fertilizer and/or soil amendment are related to its nutrient content and soil properties, which could restore overdeveloped land for garden use and increase the humus content and water-holding capacity of light-textured sand. Furthermore, SSC can be used to improve the physical and chemical properties of depleted or eroded soil^4,34,35^. The poor quality of hardened and impervious soil in the urban greenbelt is a consequence of the rapid pace of urbanization, which does not satisfy the requirement of contemporary urban greening^10^. SSC may be an excellent amendment for such urban soil^10,11,36^. Increasing evidence has indicated that SSC ploughed into the soil increases the contents of organic carbon and nutrients^4,37^. Several soil properties, such as bulk density, porosity, and water-holding capacity showed improvement because of the addition of SSC^38–40^. SSC can also play an important role in regulating soil pH^22,41^. In general, the changes in the physicochemical parameters of soil became more pronounced with increasing rates of SSC application^40^. Consistent with this trend, the pH and nutrient contents of substrates in the present study showed a significant increase with increasing application rates of SSC (Table 1). Moreover, the application of SSC significantly increased capillary capacity and total porosity, and decreased bulk density (Table 1). The physiochemical properties of the substrates of SSC treatments in this study approached the standards for growing media proposed by Hicklenton *et al*.^42^.

The positive influence of the application of SSC on soil properties generally resulted in a positive effect on the growth and nutrient uptake of a wide variety of plants^4,40,41^. Several studies have reported better growth for different woody plants following the application of SSC, including *Larix decidua* ^43^, *Paeonia suffruticosa* ^9^, and *Pinus radiata* ^44^. Consistent with these studies, our study clearly indicated that the SSC application is an effective means for improvement of *M. persiciforma* growth, with the plant height, ground diameter, and biomass of *M. persiciforma* being significantly increased by the application of SSC (Fig. 1 and Table 2). However, this growth promotion did not increase with increasing SSC application, and the best promotion occurred in S30.

Furthermore, the results of our study showed that an increasing SSC ratio was associated with a progressive increase in the accumulation of K, Cu, Zn and Cd in the roots, shoots and leaves of *M. persiciforma* (Tables 3 and 4). However, similar behaviour was not observed for N and P. The increase in heavy metal concentrations in *M. persiciforma*, particularly in the roots, resulted in concentrations of Cu and Zn in the roots in the S60 and S100 treatments that exceed the critical toxicity levels (20–30 mg·kg^−1^ for Cu and 100–300 mg·kg^−1^ for Zn)^45^. High concentrations of heavy metals, particularly Cd, Pb and Zn, can significantly disturb water and nutrient uptake, disrupt plant growth, and even result in plant death^46^. Even a moderate level of heavy metals stored in plants may cause invisible toxic symptoms^46^. Excessive application of SSC resulted in the co-existence of nutrients and excessive amounts of heavy metals, which consequently causes both positive and negative effects on plant growth. This might be an important reason for the fact that the nutrient contents in plants of the S60 and S100 treatments were much higher than those of S30, but their seedling growth and nutrient absorption was less than that of S30. The significantly higher promotion effect of SSC at an application rate of 30% compared with other application rates for *M. persiciforma* is possibly a consequence of a favourable balance between positive and negative effects.

Similar to those of Cu, Zn and Cd, the concentration of Pb in soils increased with an increasing application rate of SSC (Table 1). However, with this increase in SSC application, the Pb concentration in plants did not change accordingly (Table 4) and no significant difference was observed between the pure lateritic soil and the SSC-amended soil. The bioavailability of heavy metals depends mostly on their specific chemical fractions rather than total content^47^. McBride^48^ reported that the bonding of Pb to the SSC solids (particularly organic matter) and soil minerals generally causes very low solubility and plant uptake of Pb in SSC-amended soils. We surmised that the fact that addition of SSC in our study did not significantly increase the Pb concentration in *M. persiciforma* might be due to the bonding of Pb to SSC solids.

As reviewed by Kovacs and Szemmelveisz^46^, after plants grew successfully in heavy-metal-rich soils, every part of the plant contains heavy metal pollutants due to transport processes. The distribution of heavy metals in different organs is mainly determined by plant characteristics. Roots contact soil directly, and under normal conditions, heavy metals are mainly absorbed by, and accumulate in, the roots^49^. The accumulations of heavy metals were significantly higher in the roots than in the leaves or stems of *M. persiciforma* (Table 4).

**Figure 4:**
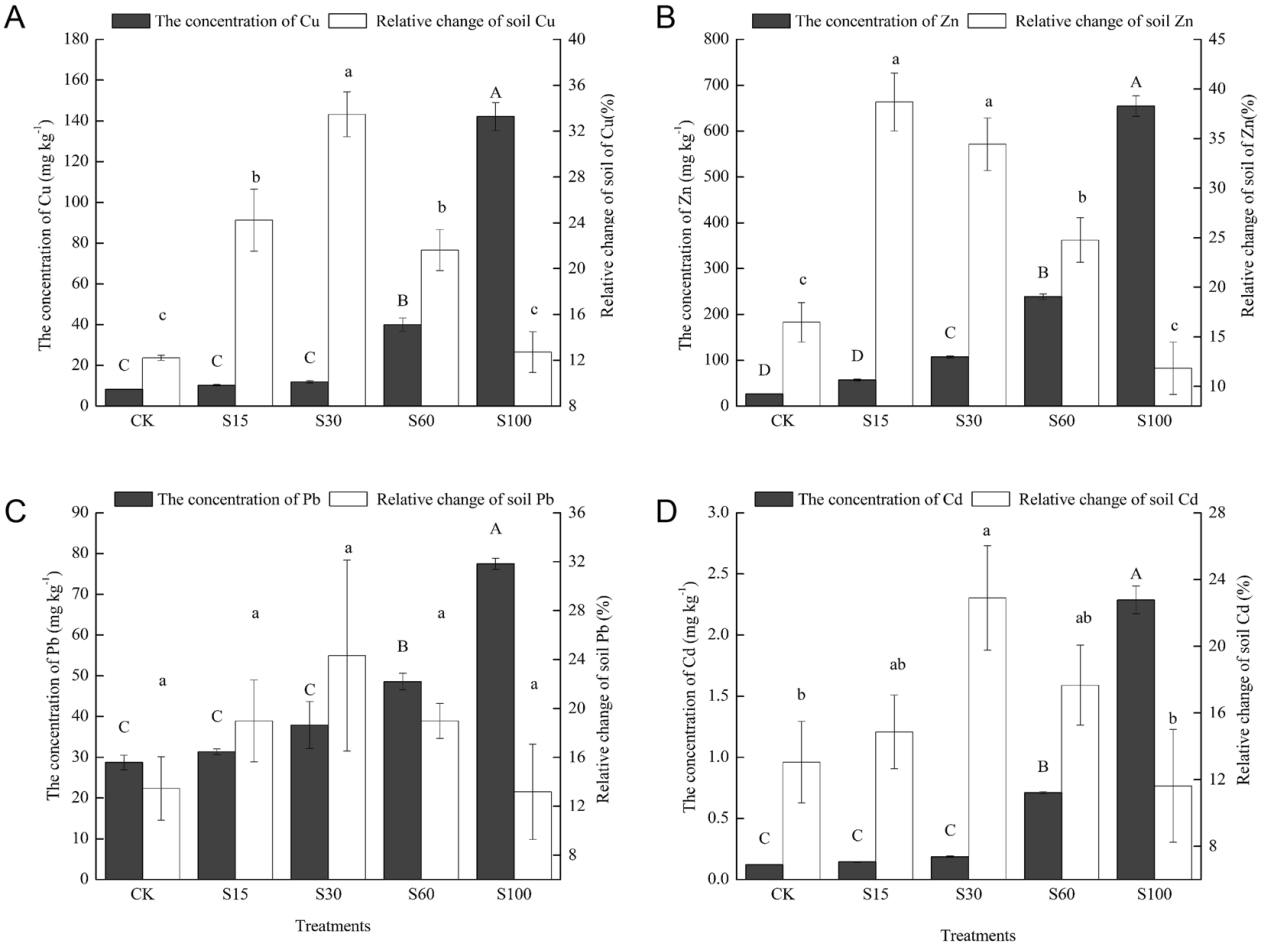
Concentrations and relative change ratio of Cu, Zn, Pb and Cd in substrates after *M. persiciforma* harvest. A, concentration and relative change ratio of Cu. B, concentration and relative change ratio of Zn. C, concentration and relative change ratio of Pb. D, concentration and relative change ratio of Cd. Values are mean ± SE (n = 5). Means with different lower-case letters are significantly different among treatments for the relative change ratio (α = 0.05 by Duncan test). Means with different upper-case letters are significantly different among treatments for the concentration (α = 0.05 by Duncan test).

Although high concentrations of heavy metals in plants may be detrimental to plant growth, some plant species are capable of growing under high concentrations of heavy metals, which has opened new possibilities for the remediation of contaminated soil. The data from Fig. 3 show that *M. persiciforma* growing on SSC-amended soil accumulated these metals in large amounts and caused appreciable reduction in heavy metal concentrations in the substrates at the end of the experiment (Fig. 4). Several other researchers, such as Suchkova *et al*.^50^, Shukla *et al*.^51^ and Macci *et al*.^52^ reported similar patterns of metal accumulation and metal reduction in various garden plants. Furthermore, the maximum Zn removal rate (38.7%) recorded in our study is higher than the Zn removal rate observed by Belhaj *et al*.^22^, who observed that the maximum rate of Zn removal by *Helianthus annuus* was 19%. Our conclusions are consistent with the suggestion by Luo *et al*.^27^, who found that woody plants with large biomass and rapid growth are more efficient than herbaceous plants in terms of heavy metal phytoremediation.

However, we found that 61.3% to 88.4% of the heavy metals still remained in the growing substrates after the 4-month potting experiment. Similar results have been reported by Belhaj *et al*.^22^ and Majid *et al*.^23^. Fortunately, when the amount of SSC applied was less than 60%, the residual contents of heavy metals in substrates were between the tier I level (the limit of the soil environmental quality to protect the natural ecology and maintain the natural environment) and the tier II level (the limit of soil quality to protect agricultural production and maintain human health) of the “Environmental Quality Standards for Soil of China” (GB 15618–1995)^33^ (Fig. 4). It is worth noting that soil amended with 30% SSC not only brought the best growth performance in *M. persiciforma* but also had residual Cu, Pb and Cd contents in substrates not significantly higher than CK after harvest. These findings suggest that SSC at reasonably low application rates can promote the growth of the landscape tree *M. persiciforma* with minimal risk of contaminating landscaping soil with heavy metals. It suggested that SSC is acceptable for large-scale utilization in landscaping, but certainly, the heavy metal removal effects must still be further verified via field-scale trials.

## Conclusions

SSC application significantly improves the fertility and water-holding capacity of landscaping soil, but it might also contaminate the soil since the SSC has high concentrations of heavy metals. We proved an optimum application rate of SSC, i.e., 30% SSC with 70% soil, could significantly increase plant height, ground diameter, and biomass of *M. persiciforma*. *M. persiciforma* was able to effectively reduce the concentrations of heavy metals (Cu, Zn, Pb and Cd) in growing substrates. A rate of 30% of SSC amendment did not pose a risk of heavy metal pollution to soil since the heavy metals were effectively removed by *M. persiciforma*. Using SSC as landscaping soil amendments and planting suitable trees such as *M. persiciforma* on amended soil could potentially provide an effective way for municipal SSC disposal and reduce fertilizer usage in urban forestry and urban greening.

## Methods

### Experimental Materials

The sewage sludge used for composting was collected from the wastewater treatment plant located in Xintang, Guangzhou, China. Sewage sludge was composted in a forced ventilation system on a pilot scale. The temperature of the compost remained above 55 °C for over a week. After 60 d of composting, the sludge compost products became loose, greyish brown and fragrant. Prior to the experiment, the SSC was spread out, air dried by repeatedly turning and mixing, sieved (2 mm) and homogenized. Lateritic soil developed from granite parent materials was collected from the arboretum of South China Agricultural University. The soil was air dried, sieved (2 mm) and homogenized. The basic properties of sewage sludge compost and soil are shown in Table 1. Plastic pots (30 cm in height, and 17 cm in diameter) were used for tree cultivation.

Healthy and uniform *M. persiciforma* seedlings were purchased from a local nursery in Guangzhou, China, with a mean height of 25.6 cm and a mean ground diameter of 4.98 mm.

### Pot Experiment Design

Lateritic soil and/or SSC were used as the growing substrate for the seedlings. Five levels of lateritic soil and/or SSC gradient, including CK (control, lateritic soil with no SSC added), S15 (15% of SSC and 85% of lateritic soil in the substrate), S30 (30% of SSC and 70% of lateritic soil in the substrate), S60 (60% of SSC and 40% of lateritic soil in the substrate) and S100 (100% of SSC and no lateritic soil in the substrate), were designed. Each treatment included 5 replicates, with a total of 25 pots in this experiment. An additional 15 pots, including 3 replicates for each treatment, were set for measuring physicochemical properties of the growing substrates. Thus, there were 40 pots in total. The mass of substrate in each pot was 3 kg on a dry mass basis. The pots were filled and left for equilibration for two weeks.

After two weeks, *M. persiciforma* seedlings were transplanted to the pots (one seedling in each pot) and watered weekly. The pots were placed in a glasshouse with the temperature set around 25 °C, a 14-h light/10-h dark photoperiod, and humidity of 85%. No pest or weed control measures were needed, and no fertilizer was applied during the experiment. The plant height and ground diameter were measured every 30 d using a ruler and a Vernier calliper, respectively.

### Sampling and Analysis

After two weeks’ equilibration, the substrates were sampled using ring samplers to determine the bulk density and porosity for each treatment. The samples for chemical analysis were collected at the same time, and then were air dried and sieved (0.25 mm). The chemical properties of the growing substrates were analysed according to Bao^53^. The pH values of the substrates were determined with a pH metre at a soil: water ratio of 1:2.5. The substrate organic matter was determined by titration after digestion with a K_2_Cr_2_O_7_-H_2_SO_4_ solution. Total nitrogen (N) was determined by the modified Kjeldahl method. Available N was determined with the alkaline hydrolysis diffusion method. Total and available phosphorus (P) were determined at a wavelength of 700 nm by the molybdenum-blue method after digestion with H_2_SO_4_-HClO_4_ and extraction with a double-acid solution (0.05 mol·L^−1^ HCl + 0.0125 mol·L^−1^ H_2_SO_4_), respectively. Total potassium (K) was determined by flame photometry on a NaOH melt, and available K was measured using 1 mol·L^−1^ NH_4_OAc extraction flame photometry.

Heavy metals in substrates before seedling planting and after seedling harvest were analysed according to the description by Bao^53^. Briefly, each substrate (1 g) was mixed with a mixture of HF-HClO_4_-HNO_3_ (i.e., 10 mL of HNO_3_, 5 mL of HClO_4_ and 5.0 mL of HF) in a polytetrafluoroethylene crucible, and allowed to stand overnight. The soil mixture was heated to 180 °C for 12 hours until the solution became clear. The sample solution was filtered into a volumetric flask and diluted to a volume of 50 mL with deionized water. The concentrations of heavy metals (Cu, Zn, Pb and Cd) were analysed via atomic absorption spectrometry (AAS).

Plants were harvested after four months. The roots, stems, and leaves of *M. persiciforma* were separated and dried to a constant weight with a thermostated drier. The dry weight of each tissue was measured using an electronic balance. The plant samples were ground with a stainless steel shredder, sieved (0.25 mm), and sealed for storage in a plastic zipper bag.

The plant samples were digested by concentrated H_2_SO_4_-H_2_O_2_ to yield a test sample solution, and the contents of N, P and K were analysed by alkaline hydrolysis diffusion, anti-colourimetric molybdenum-antimony, and flame spectrophotometry methods^50^, respectively. The stem, leaf and root samples of each plant were heated to 450 °C and digested with 1 mol·L^−1^ HNO_3_. The plant digest was filtered and transferred to a 25 mL volumetric flask. MilliQ water was used to dilute the solution to 25 mL. The contents of heavy metals (Zn, Cu, Pb and Cd) in the extracts were determined directly by atomic absorption spectrometry (AAS).

### Calculations and Statistical Analysis

The concentration of elements (nutrients and heavy metals) in the total plant (g (or mg) ·kg^−1^) (CE) is given by

1 $${\rm{CE}}=\frac{{\rm{The}}\,{\rm{accumulation}}\,{\rm{amount}}\,{\rm{of}}\,{\rm{the}}\,{\rm{elements}}}{{\rm{The}}\,{\rm{total}}\,{\rm{plant}}\,{\rm{biomass}}}$$

The accumulation amount of the elements (nutrients and heavy metals) in *M. persiciforma* (mg (or μg) ·plant^−1^) (AAE) was calculated using the following formula:

2 $${\rm{AAE}}=\sum Ci\times Mi$$

where *Ci* is the content of a certain element in one tissue of *M. persiciforma*; *Mi* is the dry weight of the organ of *M. persiciforma*.

The relative change of elements (RCE) in the substrate after seedling harvest was calculated using the following formula:

3 $${\rm{RCE}}\,( \% )=\frac{HMp-HMa}{HMp}\times 100$$

where HM_p_ and HM_a_ are the contents of the heavy metals in the substrates before and after the pot experiment, respectively.

All the experimental data were analysed using SPSS16.0. One-way analysis of variance (ANOVA) was performed on plant height, ground diameter, biomass, nutrient and heavy metal contents of *M. persiciforma* and the heavy metal contents of the substrates. If significant effects were detected (*P* < 0.05), Duncan’s multiple range test (DMRT) procedure was used to separate the mean values of each treatment at α = 0.05. Origin 8.0 was used for plotting, and the data in the figures are shown as the mean ± standard error.

## References

[1] Kelessidis A, Stasinakis AS (2012). Comparative study of the methods used for treatment and final disposal of sewage sludge in European countries. Waste Manag..

[2] Zhang QH (2016). Current status of urban wastewater treatment plants in China. Environment Int..

[3] Yue Y, Yao Y, Lin Q, Li G, Zhao X (2017). The change of heavy metals fractions during hydrochar decomposition in soils amended with different municipal sewage sludge hydrochars. J. Soil. Sediment..

[4] Bai Y, Zang C, Gu M, Gu C, Shao H (2017). Sewage sludge as an initial fertility driver for rapid improvement of mudflat salt-soils. Sci. Total Environ..

[5] Zhang L (2017). The impact of silver nanoparticles on the co-composting of sewage sludge and agricultural waste: Evolutions of organic matter and nitrogen. Bioresource Technol..

[6] Thomaidi VS, Stasinakis AS, Borova VL, Thomaidis NS (2016). Assessing the risk associated with the presence of emerging organic contaminants in sludge-amended soil: A country-level analysis. Sci. Total Environ..

[7] Tervahauta T, Rani S, Hernández Leal L, Buisman CJN, Zeeman G (2014). Black water sludge reuse in agriculture: Are heavy metals a problem?. J. Hazard. Mater..

[8] Li H, Ma Y (2016). Field study on the uptake, accumulation, translocation and risk assessment of PAHs in a soil-wheat system with amendments of sewage sludge. Sci. Total Environ..

[9] Huang X, Xue D, Xue L (2015). Changes in soil microbial functional diversity and biochemical characteristics of tree peony with amendment of sewage sludge compost. Environ. Sci. Pollut. R..

[10] Yue Y, Cui L, Lin Q, Li G, Zhao X (2017). Efficiency of sewage sludge biochar in improving urban soil properties and promoting grass growth. Chemosphere.

[11] Boen A, Haraldsen TK (2011). Fertilizer effects of increasing loads of composts and biosolids in urban greening. Urban Forest. Urban Green..

[12] Dubey RK, Simrat-Singh, Kukal SS, Kalsi HS (2013). Evaluation of different organic growing media for growth and flowering of Petunia. Commun. Soil Sci. Plant..

[13] Pathak A, Dastidar MG, Sreekrishnan TR (2009). Bioleaching of heavy metals from sewage sludge: A review. J Eenviron. Manage..

[14] Zhao S, Shang X, Duo L (2013). Accumulation and spatial distribution of Cd, Cr, and Pb in mulberry from municipal solid waste compost following application of EDTA and (NH_4_)_2_SO_4_. Environ. Sci. Pollut. R..

[15] Ali HM, EL-Mahrouk EM, Hassan FA, EL-Tarawy MA (2011). Usage of sewage effluent in irrigation of some woody tree seedlings. Part 3: *Swietenia mahagoni* (L.) Jacq. Saudi. Saui. J. Biol. Sci..

[16] Tai Y, Li Z, Mcbride MB (2016). Natural attenuation of toxic metal phytoavailability in 35-year-old sewage sludge-amended soil. Environ. Monit. Assess..

[17] Yeganeh M, Afyuni M, Khoshgoftarmanesh AH, Rezaeinejad Y, Schulin R (2011). Transport of zinc, copper, and lead in a sewage sludge amended calcareous soil. Soil Use Manage..

[18] Fang W, Delapp RC, Kosson DS, van der Sloot HA, Liu J (2017). Release of heavy metals during long-term land application of sewage sludge compost: Percolation leaching tests with repeated additions of compost. Chemosphere.

[19] Liu H (2016). Achilles heel of environmental risk from recycling of sludge to soil as amendment: A summary in recent ten years (2007–2016). Waste Manag..

[20] Kumar V, Chopra AK (2014). Accumulation and translocation of metals in soil and different parts of french bean (*Phaseolus vulgaris* L.) amended with sewage sludge. B. Environ. Contam. Tox..

[21] De Lucia B, Cristiano G, Vecchietti L, Bruno L (2013). Effect of different rates of composted organic amendment on urban soil properties, growth and nutrient status of three Mediterranean native hedge species. Urban For. Urban Gree..

[22] Belhaj D (2016). Effects of sewage sludge fertilizer on heavy metal accumulation and consequent responses of sunflower (*Helianthus annuus*). Environ. Sci. Pollut. Res..

[23] Majid NM, Islam MM, Riasmi Y, Abdu A (2012). Assessment of heavy metal uptake and translocation by *Pluchea indica* L. from sawdust sludge contaminated soil. J. Food Agric. Environ..

[24] Majid NM, Islam MM, Enanee N (2012). Heavy metal uptake and translocation by semuloh *(Fagopyrum dibotrys*) from sawdust sludge contaminated soil. Bulg. J. Agric. Sci..

[25] Kumwimba MN, Zhu B, Suanon FL, Muyembe DK, Dzakpasu M (2017). Long-term impact of primary domestic sewage on metal/loid accumulation in drainage ditch sediments, plants and water: Implications for phytoremediation and restoration. Sci. Total Environ..

[26] van der Ent A, Baker AJM, Reeves RD, Pollard AJ, Schat H (2013). Hyperaccumulators of metal and metalloid trace elements: Facts and fiction. Plant Soil.

[27] Luo Z, He J, Polle A, Rennenberg H (2016). Heavy metal accumulation and signal transduction in herbaceous and woody plants: Paving the way for enhancing phytoremediation efficiency. Biotechnol. Adv..

[28] Yakun S, Xingmin M, Kairong L, Hongbo S (2016). Soil characterization and differential patterns of heavy metal accumulation in woody plants grown in coal gangue wastelands in Shaanxi, China. Environ. Sci. Pollut. R..

[29] Kub Tov P, Hejcman M, Sz Kov JI, Vondr Kov S, Tlusto P (2016). Effects of sewage sludge application on biomass production and concentrations of Cd, Pb and Zn in shoots of *Salix* and *Populus* clones: Improvement of phytoremediation efficiency in contaminated soils. Bio. Energ. Res..

[30] Urbaniak M, Wyrwicka A, Tołoczko W, Serwecińska L, Zieliński M (2017). The effect of sewage sludge application on soil properties and willow (*Salix sp*.) cultivation. Sci. Total Environ..

[31] Houda Z, Bejaoui Z, Albouchi A, Gupta DK, Corpas FJ (2016). Comparative study of plant growth of two poplar tree species irrigated with treated wastewater, with particular reference to accumulation of heavy metals (Cd, Pb, As, and Ni). Environ Monit Assess..

[32] Qin J, Deng J, Feng X, Wang Q, Wang S (2008). Quantitative RP–LC analysis of mangiferin and homomangiferin in *Mangifera indica L*. leaves and in *Mangifera persiciforma* C.Y. Wu et T.L. Ming Leaves. Chromatographia..

[33] Ministry of Environmental Protection of the People’s Republic of China. Environmental Quality Standards for Soils of China (GB15618-1995). http://kjs.mep.gov.cn/hjbhbz/bzwb/trhj/trhjzlbz/199603/t19960301_82028.shtml (1996).

[34] Zhang J (2015). Multiscale visualization of the structural and characteristic changes of sewage sludge biochar oriented towards potential agronomic and environmental implication. Sci. Rep..

[35] Clarke BO, Smith SR (2011). Review of ‘emerging’ organic contaminants in biosolids and assessment of international research priorities for the agricultural use of biosolids. Environ. Int..

[36] Ma W (2015). Environmental evaluation of the application of compost sewage sludge to landscaping as soil amendments: a field. Desalin. Water Treat..

[37] Zoghlami RI, Hamdi H, Mokni-Tlili S, Khelil MN, Aissa NB (2016). Changes in light-textured soil parameters following two successive annual amendments with urban sewage sludge. Ecol. Eng..

[38] Song U, Lee EJ (2010). Environmental and economical assessment of sewage sludge compost application on soil and plants in a landfill. Resour. Conserv. Recy..

[39] Sharma B, Sarkar A, Singh P, Singh RP (2017). Agricultural utilization of biosolids: A review on potential effects on soil and plant grown. Waste Manag..

[40] Xue D, Huang X (2013). The impact of sewage sludge compost on tree peony growth and soil microbiological, and biochemical properties. Chemosphere.

[41] Mosquera-Losada MR, Ferreiro-Domínguez N, Daboussi S, Rigueiro-Rodríguez A (2016). Sewage sludge stabilisation and fertiliser value in a silvopastoral system developed with *Eucalyptus nitens* Maiden in Lugo (Spain). Sci. Total Environ..

[42] Hicklenton PR, Rodd V, Warman PR (2001). The effectiveness and consistency of source-separated municipal solid waste and bark composts as components of container growing media. Sci. Hortic..

[43] Bourioug M (2015). Sewage sludge fertilization in larch seedlings: Effects on trace metal accumulation and growth performance. Ecol. Eng..

[44] Ferreiro-Domínguez N, Rigueiro-Rodríguez A, Mosquera-Losada MR (2012). Sewage sludge fertiliser use: Implications for soil and plant copper evolution in forest and agronomic soils. Sci. Total Environ..

[45] Maestri E, Marmiroli M, Visioli G, Marmiroli N (2010). Metal tolerance and hyperaccumulation: Costs and trade-offs between traits and environment. Environ. Exp. Bot..

[46] Kovacs H, Szemmelveisz K (2017). Disposal options for polluted plants grown on heavy metal contaminated brown field lands? A review. Chemosphere.

[47] Singh J, Kalamdhad AS (2013). Effect of *Eisenia fetida* on speciation of heavy metals during vermin composting of water hyacinth. Ecol. Eng..

[48] McBride MB (2003). Toxic metals in sewage sludge-amended soils: has promotion of beneficial use discounted the risks?. Adv. Environ. Res..

[49] Soriano-Disla JM, Gómez IO, Navarro-Pedreño J, Jordán MM (2014). The transfer of heavy metals to barley plants from soils amended with sewage sludge with different heavy metal burdens. J. Soil. Sediment..

[50] Suchkova N (2014). Assessment of phytoremediation potential of native plants during the reclamation of an area affected by sewage sludge. Ecol. Eng..

[51] Shukla OP, Juwarkar AA, Singh SK, Khan S, Rai UN (2011). Growth responses and metal accumulation capabilities of woody plants during the phytoremediation of tannery sludge. Waste Manag..

[52] Macci C, Peruzzi E, Doni S, Iannelli R, Masciandaro G (2015). Ornamental plants for micropollutant removal in wetland systems. Environ. Sci. Pollut. R..

[53] Bao, S. D. *Methods for Soil Agricultural and Chemical Analysis*, 263–282 (China Agriculture Press, Beijing, 2000).

